# Disease-Based Criteria vs BMI Level for Prioritization of Metabolic Surgery

**DOI:** 10.1007/s11695-025-07896-4

**Published:** 2025-05-17

**Authors:** Pia Roser, Robert D. McIntyre, Simone Cremona, Adel Assiri, Lyz Bezerra Silva, Ghassan Chamseddine, Francesco Rubino

**Affiliations:** 1 University Medical Center Hamburg-Eppendorf, Hamburg, Germany; 2 King’s College London, London, United Kingdom; 3 St Mary’s University Twickenham, London, United Kingdom; 4 Hospital Del Mar, Barcelona, Spain

**Keywords:** Bariatric surgery, Metabolic surgery, Obesity, Type 2 diabetes, Cardiovascular disease, BMI

## Abstract

**Background:**

BMI is widely used as a primary criterion for prioritizing candidates for metabolic surgery. However, it may not fully capture disease severity or mortality risks associated with comorbidities such as type 2 diabetes (T2D) and cardiovascular disease (CVD). This study aimed to assess whether BMI accurately reflects disease burden and risk in patients undergoing metabolic surgery.

**Methods:**

A retrospective audit included 723 adult candidates for primary metabolic surgery at a tertiary care center between January 2014 and December 2022. Patients undergoing revisional surgeries were excluded. Clinical data, including demographics, comorbidities, and disease severity indicators (e.g., ASA score, Charlson Comorbidity Index [CCI], medication usage, and estimated 10-year survival), were analyzed. Patients were grouped by BMI (< or ≥ 50 kg/m^2^), T2D, and CVD status for comparison.

**Results:**

Prevalence rates for T2D, BMI ≥ 50 kg/m^2^, and CVD were 41.6%, 37.3%, and 16.2%, respectively. Patients with BMI ≥ 50 kg/m^2^ were generally younger, had fewer comorbidities, lower CVD prevalence, and better estimated 10-year survival than those with BMI < 50 kg/m^2^. In contrast, patients with T2D and CVD had significantly higher ASA and CCI scores, greater medication usage, and reduced 10-year survival (*p* < 0.001 for T2D; *p* < 0.01 for CVD).

**Conclusion:**

Higher BMI levels do not reflect greater disease burden and mortality risk among candidates for bariatric/metabolic surgery. These findings do not support the use of high BMI-based thresholds (e.g., ≥ 50 kg/m^2^) as criteria for expedited access. Clinically relevant measures of baseline disease burden should be used to determine the urgency of access to surgical treatment of obesity and T2D.

## Introduction

Originally conceived as a mere weight loss intervention to reduce obesity-related health risk, bariatric surgery has proven to be a highly effective treatment for various diseases and conditions, most notably type 2 diabetes (T2D) [1].

Mechanistic studies show that gastrointestinal operations improve glucose metabolism by weight-independent mechanisms [2]. Evidence from numerous clinical studies assessing the effects of surgery in patients with T2D, including several randomized clinical trials, show that bariatric surgery can achieve durable remission of hyperglycemia [1], dramatic reduction of cardiometabolic risk factors and diabetes-related complications [3], as well as substantial mortality reduction, both diabetes-related and overall [4]. Based on such biological and clinical evidence, bariatric surgery has been increasingly used to treat patients with T2D, a practice referred to as metabolic surgery. Bariatric and metabolic surgery is also highly effective at achieving long-term control of hypertension [5], improved renal function [6] and metabolic dysfunction-associated steatohepatitis (MASH)-related fibrosis [7]. Several studies show that bariatric and metabolic surgery is more cost-effective [8] and has greater life-extending potential in patients with T2D at baseline, compared to patients without diabetes [9].

The increased prevalence of T2D among candidates for bariatric and metabolic surgery has led to substantial changes in patients’ demographics and baseline characteristics compared to traditional bariatric surgery practices [10]. In fact, surgical candidates with T2D are generally older, have higher number of co-morbidities and previous cardiovascular disease (CVD) compared to surgical candidates without diabetes [8].

Despite such transformative changes in the patient population now being considered for bariatric and metabolic surgery, body mass index (BMI)-based eligibility criteria for patients’ selection, assessment of treatment urgency, and prioritization of access to surgery remain outdated and increasingly misaligned with the current clinical reality. In fact, BMI is not a measure of health or illness at the individual level as it provides no information about the functioning of organs and tissues [11]. Thus, BMI levels cannot be used as a measure of ongoing disease or disease severity. The health risk associated with BMI also varies substantially with age and ethnicity [12, 13]. In many countries, and especially in Asia, individuals with T2D have mean BMI levels lower than 35 kg/m^2^; thus, using BMI-thresholds for surgical indication can effectively exclude many patients from potential surgical benefit [14, 15]. These findings from prior research emphasize the limitations of BMI-centric guidelines and highlight the need for more inclusive and clinically relevant eligibility criteria. Yet, health insurance coverage of bariatric and metabolic surgery is still universally based on BMI thresholds, and criteria for insurance coverage often require surgical candidates to undergo non-surgical pre-operative weight management (e.g., “Tier 3” in UK ([16]).

For patients who meet BMI-centric criteria for surgical indication, access to bariatric and metabolic surgery, especially in publicly funded healthcare systems, is often granted on a first-come-first-served basis, regardless of disease severity [17]. These practices may effectively delay treatment in many patients who are at higher risk of disease progression and significant, yet preventable complications.

In many healthcare systems including, as an example, the UK’s NHS (National Health Service) expedited access to bariatric and metabolic surgery is reserved only for people with very high BMI (e.g., ≥ 50 kg/m^2^) [16]; it is unclear, however, if such BMI threshold accurately reflects disease burden or treatment urgency in candidates to metabolic surgery.

In this study, we used an audit of our clinical practice to investigate demographic characteristics, disease severity, and disease-related mortality risk among candidates for bariatric and metabolic surgery, according to diabetes status, the presence of established CVD or BMI ≥ 50 kg/m^2^ at baseline.

## Methods

### Study Design

**Fig. 1:**
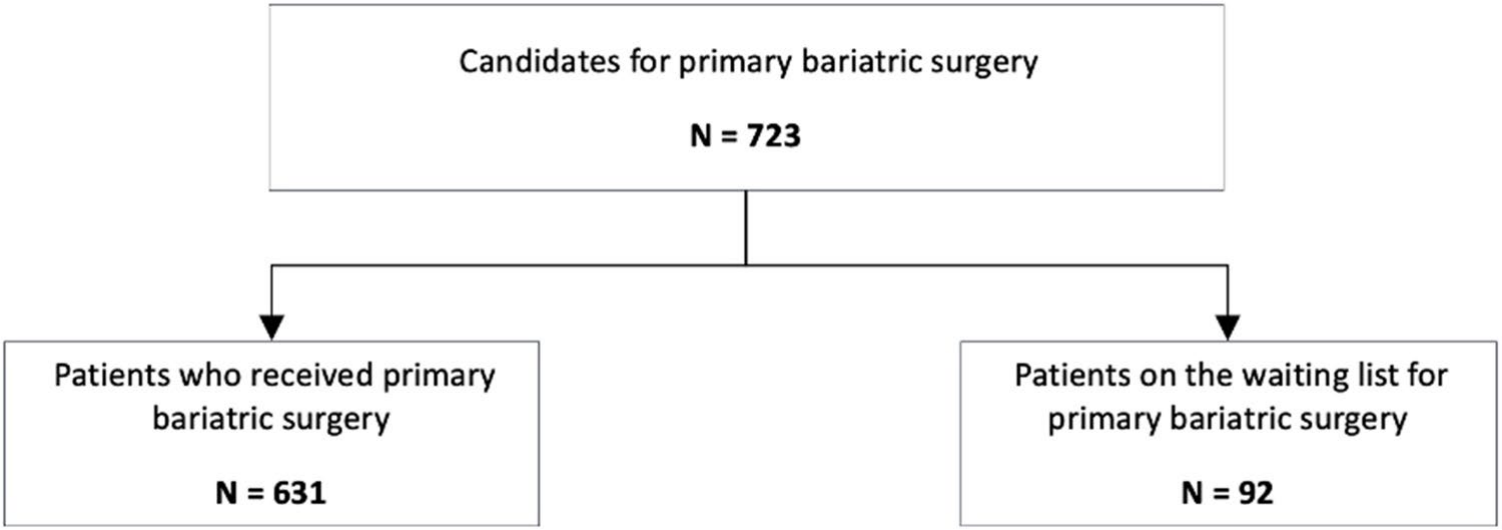
Flow chart of patient selection. Flow diagram of the cases included in analysis

For this investigation, we performed an audit of a single consultant surgeon’s practice at a tertiary care center (King´s College Hospital, NHS Foundation Trust in London, UK). The audit analyzed data from an anonymized database with peri-operative data from clinical records of patients who routinely underwent bariatric and metabolic surgery between January 2014 and December 2022 or were on the waiting list for surgery at the time of this analysis. Criteria for inclusion in the specific analysis of this report included an age of 18 years or above and primary bariatric and metabolic surgery. Exclusion criteria specifically excluded cases of revisional bariatric procedures, including band removal and other operations for weight regain or complications of previous procedures. A flow diagram of the patient selection is presented in Fig. 1.

To investigate whether BMI accurately reflects disease burden and surgical risk, patients were stratified into three key comparison groups:

1. Presence vs. absence of T2D
2. Presence vs. absence of CVD
3. BMI ≥ 50 kg/m^2^ vs. BMI < 50 kg/m^2^

We compared demographics, baseline clinical characteristics, indicators of disease severity at baseline, and peri-operative safety outcomes across the subgroups. All surgeries were performed by a single consultant-level surgeon (FR) as the primary operator.

This report presents the results of an audit of routine clinical practice and is based on an anonymized, ongoing database maintained to monitor characteristics of surgical candidates and peri-operative outcomes. In accordance with policies and advice of the local clinical governance office, this study did not require formal research ethics committee approval.

### Patient and Public Involvement

Patients and the public were not directly involved in the design, recruitment, or conduct of this retrospective clinical audit, as the study utilized pre-existing clinical data from standard practice. Since this study did not require specific patient recruitment, nor the use of new interventions, patient input on the burden of participation or intervention timing was not applicable. As noted above, ethical approval was not required as this was a retrospective audit of a database maintained for the purpose of service evaluation.

### Main Outcome Measures

To assess the significance of BMI level vs clinical characteristics (diabetes status and previous CVD), we compared patients’ demographics and several baseline measures of disease severity in patients with BMI ≥ 50 vs < 50 kg/m^2^ as well as in patients with vs without pre-operative T2D or with vs without CVD.

Measures of disease severity included the Charlson Comorbidity Index (CCI) score, estimated 10-year survival, American Society of Anesthesiologists (ASA) score, overall number of co-morbidities, and medication usage. Additionally, we assessed safety measures among operated patients according to the above baseline disease status. Safety measures included hospital length of stay (LoS), in-patient complications, 30-day-major complications, and readmission rates.

### Data Collection and Definitions

We analyzed age and gender differences among groups as recorded in our standard clinical practice. The overall number of “co-morbidities” was measured based on the presence of one or more obesity-related diseases and conditions. These included T2D, hypertension, dyslipidemia, CVD, metabolic dysfunction-associated steatohepatitis/metabolic dysfunction-associated fatty liver disease (MASH/MAFLD), chronic kidney disease (CKD), obstructive sleep apnea syndrome (OSAS), osteoarthritis, and polycystic ovary syndrome (PCOS). The data entered in our database derive from routinely used, standardized electronic medical records and pre-surgical assessment forms conducted by a multidisciplinary bariatric team. This ensures consistent identification and recording of baseline characteristics and comorbidities. CVD was defined as a documented pre-operative history of significant cardiovascular diseases and conditions, including previous myocardial infarction, atrial fibrillation, angina pectoris, arrhythmia, coronary artery disease, cerebrovascular accident, cardiomyopathy, heart failure, left ventricular dysfunction, pulmonary hypertension, WPW (Wolff–Parkinson–White) syndrome, transient ischemic attack, stroke, or peripheral vascular disease. Diabetes status and other metabolic diseases were assessed from documented medical histories or as newly diagnosed disease. New onset diabetes was diagnosed preoperatively based on a glycated hemoglobin level of 6.5% or higher or a fasting glucose level of 126 mg/dL or higher or 2-h plasma glucose levels ≥ 200 mg/dL during OGTT [18]. Newly diagnosed dyslipidemia adhered to the European Society of Cardiology (ESC) Guidelines [19], indicating a low-density lipoprotein cholesterol (LDL-C) level of 116 mg/dL (3.0 mmol/L) or above and a triglyceride (TG) level of 177 mg/dL (2.0 mmol/L) or above. Pharmacological treatment of metabolic diseases, including diabetes, dyslipidemia, hypertension, and depression, was meticulously recorded for each patient in our standard practice; hence, reliable data about baseline obesity-related medication usage was available for analysis.

Operative risk assessment is a measure of baseline disease severity. For such assessment, we used the ASA Classification [20, 21], where ASA class 1 indicates a healthy patient and ASA class 2 indicates a patient with mild systemic disease. The individual risk of death from baseline disease(s) was assessed using the CCI [22]. Based on the original publication of Charlson et al. [17] and in line with a publication using similar thresholds by Huang et al. [23], patients were categorized into two groups according to their mortality risk: a “low-risk group” with CCI scores less than 3 and a “high-risk group” with CCI scores of 3 or higher.

The estimated 10-year survival rate was calculated using the following equation: Estimated 10-year survival = 0.983^(eCCI × 0.9) [22].

Peri-operative safety measures included the LoS, inpatient complication rate, 30-day major complications’ rate, and 30-day readmissions’ rate. Surgical complications were defined according to the Clavien-Dindo classification criteria [24]. Accordingly, major surgical complications were defined as Clavien-Dindo’s level ≥ III indicating complications requiring procedural intervention (surgical, radiological or endoscopic) or use of intensive care.

### Statistical Analysis

Data were analyzed using SPSS version 27 (IBM Corporation, NY, USA) or GraphPad Prism Version 9.1 (GraphPad Software Inc. La Jolla, USA). Data that were non-normally distributed were reported as median and interquartile range (IQR), unless otherwise stated. Statistical significance was accepted at *p* < 0.05. Patient characteristics and perioperative outcomes were compared using the Mann–Whitney *U* test for ratio variables or chi-square test for categorical variables.

## Results

### Baseline Characteristics

#### Comparison of Patients with vs. Without T2D

Among the 723 patients eligible for analysis, 301 (41.6%) had baseline T2D. Notably, individuals with T2D were characterized by an older age (51 ± 11 vs 45 ± 12; *p* < 0.001) and a lower BMI (47 ± 8 vs 49 ± 8; *p* < 0.001) compared to patients without diabetes (Table 1).

**Table 1:** Summary of clinical characteristics in all patients and patients with vs. without T2D

Clinical characteristics	All patients	Type 2 diabetes	No diabetes	*p*-value
	*n* = 723	*n* = 301 (41.6%)	*n* = 422 (58.4%)	
Age (years)	47 ± 12	51 ± 11	45 ± 12	*p* < 0.001
Gender, female — no. (%)	518 (71.6%)	192 (63.8)	326 (77.3)	*p* < 0.001
BMI (kg/m^2^)	48 ± 8	47 ± 8	49 ± 8	*p* < 0.001
CCI score	1.6 ± 1.6	2.5 ± 1.7	0.8 ± 1.1	*p* < 0.001
Estimated 10-year survival (%)	93.0	85.0	96.5	*p* < 0.001
ASA score	2.6 ± 0.5	2.7 ± 0.5	2.5 ± 0.6	*p* < 0.001
Number of comorbidities	3.8 ± 2.3	4.9 ± 2.1	3.0 ± 2.1	*p* < 0.001
Number of medications	1.7 ± 2.1	3.3 ± 2.1	0.6 ± 1.0	*p* < 0.001
BMI ≥ 50 — no. (%)	270 (37.3%)	102 (33.9)	167 (39.8)	*p* = 0.105
CVD — no. (%)	117 (16.2%)	68 (22.6)	49 (11.6)	*p* < 0.001

Continuous data are presented as mean ± SD and anlayzed by two-sided *T*-test. Categorical data are presented as count (%) and analyzed by Pearson’s chi-square test. *BMI* body mass index, *CCI* Charlson Comorbidity Index, *ASA* American Society of Anaesthesiologists, *CVD* cardiovascular disease

The T2D group also had a statistically significantly higher CCI (2.5 ± 1.7 vs 0.8 ± 1.1; *p* < 0.001) and ASA score (2.7 ± 0.5 vs 2.5 ± 0.6; *p* < 0.001) indicating increased baseline disease burden. They also had a higher number of comorbidities (4.9 ± 2.1 vs 3.0 ± 2.1; *p* < 0.001) and were taking more medications (3.3 ± 2.1 vs 0.6 ± 1.0; *p* < 0.001). The prevalence of CVD was also significantly higher in this group (22.6% vs 11.6%; *p* < 0.001). Furthermore, the estimated 10-year survival rate was lower in patients with T2D compared to patients without diabetes (85.0% vs 96.5%; *p* < 0.001). Among patients without T2D, there was a higher proportion of individuals with BMI ≥ 50 kg/m^2^ (39.8% vs 33.9%), although this difference did not reach statistical significance (*p* = 0.105). When adjusted for age, the differences between the two subgroups remained statistically significant for all variables except for BMI (*p* = 0.056).

#### Comparison of Patients with BMI ≥ 50 vs < 50 kg/m.^2^

At baseline, 270 patients (37.3%) had a BMI ≥ 50 kg/m^2^. As shown in Table 2, patients with BMI < 50 kg/m^2^ were older (49 ± 11 vs 44 ± 12 years; *p* < 0.001) and had more comorbidities (3.9 ± 2.3 vs 3.6 ± 2.1; *p* = 0.041), higher CCI scores (1.7 ± 1.7 vs 1.4 ± 1.4; *p* = 0.011), and more medications (1.9 ± 2.2 vs 1.3 ± 1.7; *p* < 0.001).

**Table 2:** Summary of baseline clinical characteristics in patients with BMI ≥ 50 vs. BMI < 50 kg/m^2^

Clinical characteristics	BMI ≥ 50	BMI < 50	*p*-value
	*n* = 270 (37.3%)	*n* = 453 (62.7%)	
Age (years)	44 ± 12	49 ± 11	*p* < 0.001
Gender, female — no. (%)	193 (71.5)	325 (71.7)	*p* = 0.940
BMI (kg/m^2^)	56 ± 7	43 ± 4	*p* < 0.001
CCI score	1.4 ± 1.4	1.7 ± 1.7	*p* = 0.011
Estimated 10-year survival (%)	94.1	92.4	*p* = 0.011
ASA score	2.6 ± 0.5	2.5 ± 0.6	*p* = 0.003
Number of comorbidities	3.6 ± 2.1	3.9 ± 2.3	*p* = 0.041
Number of medications	1.3 ± 1.7	1.9 ± 2.2	*p* < 0.001
Diabetes — no. (%)	102 (37.8)	199 (43.9)	*p* = 0.105
CVD — no. (%)	31 (11.5)	86 (19.0)	*p* = 0.008

Continuous data are presented as mean ± SD and analyzed by two-sided *T*-test. Categorical data are presented as count (%) and analyzed by Pearson’s chi-square test. *BMI* body mass index, *CCI* Charlson Comorbidity Index, *ASA* American Society of Anaesthesiologists, *CVD* cardiovascular disease

Additionally, CVD was more prevalent in patients with BMI < 50 kg/m^2^ (19.0% vs 11.5%; *p* = 0.008). However, there was no statistically significant difference in T2D prevalence (*p* = 0.105) between the two groups. The estimated 10-year survival was lower in the BMI < 50 kg/m^2^ group (92.4% vs 94.1%; *p* = 0.011).

When adjusted for age, most differences became non-significant, suggesting that age, rather than BMI, may better reflect disease severity. These findings reinforce that a BMI ≥ 50 kg/m^2^ is not a reliable marker of disease burden or treatment urgency.

#### Comparison of Groups with vs Without Baseline CVD

Of all patients, 117 (16.2%) had CVD at baseline. This group was significantly older (55 ± 10 vs 46 ± 12 years; *p* < 0.001), had a lower BMI (46 ± 7 vs 48 ± 8 kg/m^2^; *p* = 0.003), and exhibited higher CCI (3.1 ± 2.0 vs 1.3 ± 1.4; *p* < 0.001) and ASA scores (2.8 ± 0.4 vs 2.5 ± 0.6; *p* < 0.001) (Table 3).

**Table 3:** Summary of clinical characteristics in patients with vs. without CVD

Clinical characteristics	CVD	No CVD	*p*-value
	*n* = 117 (16.2%)	*n* = 606 (83.8%)	
Age (years)	55 ± 10	46 ± 12	*p* < 0.001
Gender, female — no. (%)	64 (54.7)	454 (74.9)	*p* < 0.001
BMI (kg/m^2^)	46 ± 7	48 ± 8	*p* = 0.003
CCI score	3.1 ± 2.0	1.3 ± 1.4	*p* < 0.001
Estimated 10-year survival (%)	75.6	94.6	*p* < 0.001
ASA score	2.8 ± 0.4	2.5 ± 0.6	*p* < 0.001
Number of comorbidities	6.0 ± 2.4	3.4 ± 2.0	*p* < 0.001
Number of medications	3.0 ± 2.4	1.5 ± 1.9	*p* < 0.001
Diabetes — no. (%)	68 (58.1)	233 (38.4)	*p* < 0.001
BMI ≥ 50 — no. (%)	31 (26.5)	239 (39.4)	*p* = 0.008

Continuous data are presented as mean ± SD and analyzed by two-sided *T*-test. Categorical data are presented as count (%) and analyzed by Pearson’s chi-square test. *BMI* body mass index, *CCI* Charlson Comorbidity Index, *ASA* American Society of Anaesthesiologists

They also had a greater number of comorbidities (6.0 ± 2.4 vs 3.4 ± 2.0; *p* < 0.001) and higher medication use (3.0 ± 2.4 vs 1.5 ± 1.9; *p* < 0.001). T2D prevalence was significantly higher in patients with CVD (58.1% vs 38.4%; *p* < 0.001), while a lower proportion had BMI ≥ 50 kg/m^2^ (26.5% vs 39.4%; *p* = 0.008).

The estimated 10-year survival was considerably lower in patients with CVD (75.6% vs 94.6%; *p* < 0.001). After adjusting for age, all differences, except for BMI (*p* = 0.20), remained statistically significant.

### Comparative Findings of Disease Severity Indicators by BMI, T2D, and CVD Status

**Fig. 2:**
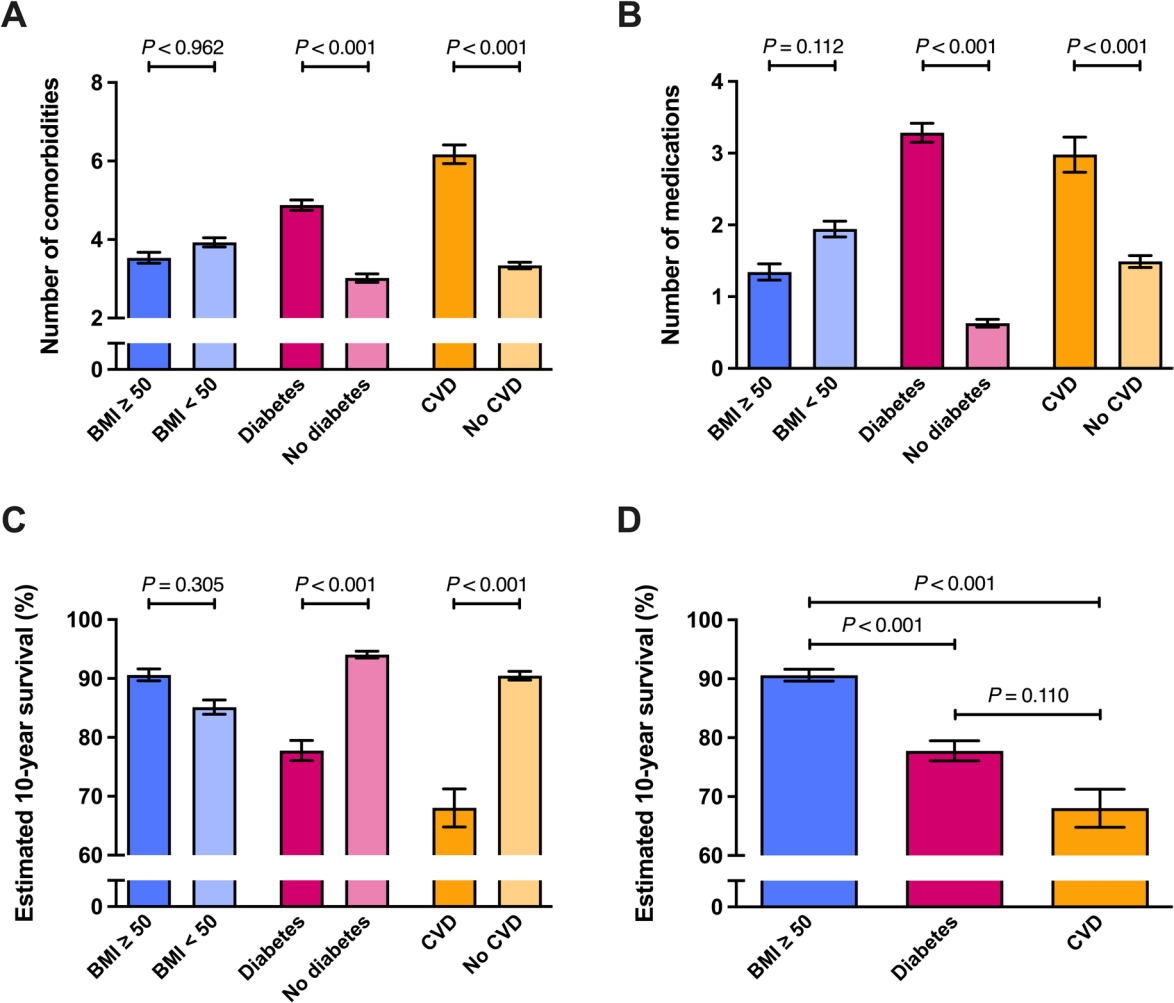
**A–D** Comparison of comorbidities, medication usage, and estimated 10-year survival by BMI, T2D, and CVD status. Legend: Bars represent mean ± SE number of comorbidities (**A**), number of medications (**B**), or estimated 10-year survival (**C**, **D**) in patients with diabetes, without diabetes, with a BMI ≥ 50, with a BMI < 50, with cardiovascular disease (CVD), or without CVD. Analyzed by one-way ANCOVA with age as a covariate. **D** Comparison has been analyzed by a one-way ANCOVA and post-hoc pairwise comparisons with Bonferroni correction factor. *The subgroup BMI ≥ 50 in **D** includes patients with diabetes and CVD

Figure 2 illustrates the comparative analysis of disease severity indicators, including the number of comorbidities and medications as well as the estimated 10-year survival among subgroups stratified by BMI, T2D, and CVD status.

#### Comparison of Comorbidities, Medication Usage, and Estimated 10-Year Survival by BMI, T2D, and CVD Status (Fig. 2A–D***)***

Figure 2A shows that while there was no significant difference in the number of comorbidities between patients with BMI ≥ 50 and BMI < 50 kg/m^2^ (*p* = 0.962), patients with baseline T2D or established CVD had significantly more comorbidities compared to their respective counterparts (both *p* < 0.001).

Figure 2B demonstrates that the number of medications taken by patients did not differ significantly between BMI subgroups (*p* = 0.112). However, patients with baseline T2D or CVD required significantly more medications than those without these conditions (*p* < 0.001 for both comparisons).

Figure 2C depicts estimated 10-year survival rates. Patients with T2D or CVD had significantly lower estimated survival compared to those without T2D or CVD (both *p* < 0.001). Conversely, the difference in estimated 10-year survival between BMI groups (≥ 50 vs. < 50 kg/m^2^) was not significant (*p* = 0.305).

Figure 2D compares estimated 10-year survival directly across the three high-risk groups, revealing that patients with CVD had the lowest estimated survival, significantly lower compared to patients with T2D (*p* < 0.001) or a BMI ≥ 50 kg/m^2^ (*p* < 0.001). The difference between the T2D and BMI ≥ 50 kg/m^2^ groups, however, was not statistically significant (*p* = 0.110).

These findings suggest that T2D and CVD status are stronger indicators of disease burden, medication usage, and estimated 10-year survival rate than BMI alone, emphasizing the limitations of using BMI thresholds to determine surgical urgency and eligibility.

### Perioperative Safety Outcomes

The safety outcomes of primary bariatric and metabolic surgery were assessed in the 631 operated patients. The average LoS was 2.8 ± 1.5 days. The rate of major inpatient complications was low at 0.8% (*n* = 5), with a 30-day major complication rate of 1.6% (*n* = 10) and a 30-day readmission rate of 4.9% (*n* = 31).

Patients with T2D had a slightly but statistically significant longer LoS compared to patients without diabetes (3.0 ± 1.4 days vs 2.6 ± 1.5 days; *p* = 0.002); there were no significant LoS differences between groups with vs. without CVD and with or by BMI category.

However, patients with CVD had a significantly higher 30-day-readmission rate when compared to patients without CVD (11.1% vs 3.8%; *p* = 0.002). Patients with BMI ≥ 50 kg/m^2^ showed a non-significant trend toward a higher 30-day complication rate *(p* = 0.324).

**Fig. 3:**
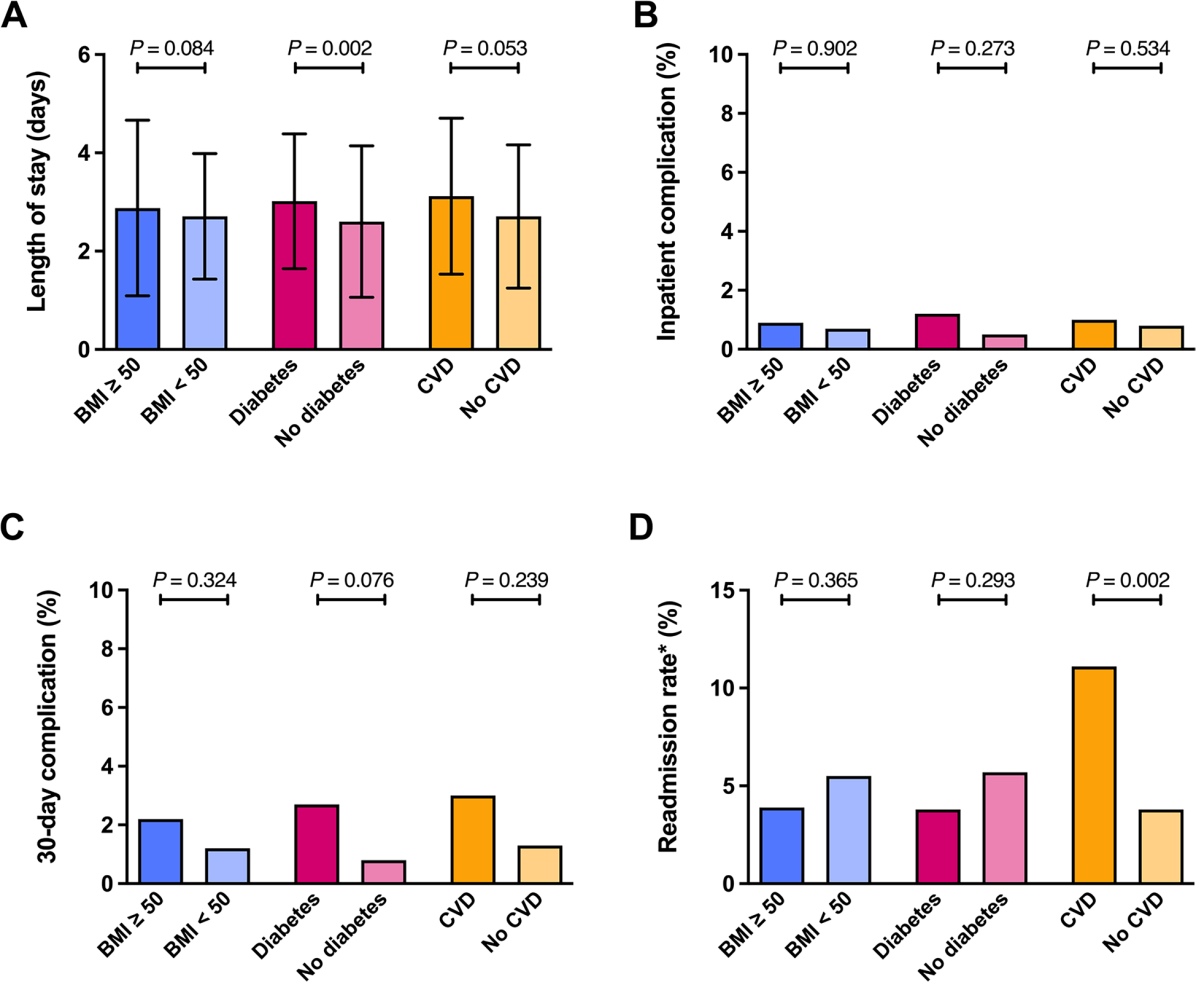
Perioperative safety outcomes. Legend: Bars represent mean ± SE length of hospital stay (LoS) (**A**), the proportion of inpatient complications (**B**), the proportion of 30-day complications (**C**), or readmission rate *(all causes) within 30 days of bariatric surgery (**D**) in patients with diabetes, without diabetes, with a BMI ≥ 50 kg/m^2^, with a BMI < 50 kg/m^2^, with cardiovascular disease (CVD), or without CVD. LoS was analyzed by one-way ANCOVA, and complications and readmission rate were analyzed by binary logistic regression with gender, age, and BMI as covariates

No other significant differences in safety outcomes were observed across subgroups. Figure 3A–D provides a visual summary of perioperative safety outcomes by subgroup.

## Discussion

The results of this study provide a real-world picture of the characteristics of the patient population of modern metabolic surgery and highlight the clinically significant differences with published series of traditional bariatric surgery. Compared to large series from traditional bariatric surgery [25, 26], the patient population in this audit of metabolic surgery is older (mean age = 47 years in this series vs a range of 39–45 years in published series of bariatric surgery [25, 26]), has a greater prevalence of male gender (28% in this series vs 19% in bariatric surgery series [25, 26]), T2D (41% in this series vs range 15–36% in reported series of traditional bariatric surgery [25, 26]), and CVD (16% in this series vs approximately 7% in the largest series of bariatric surgery [26]). Our data are in line with baseline characteristics from other recent series of metabolic surgery [27–29]. This audit also demonstrates that baseline diabetes and CVD status are far more reliable measures of disease severity and treatment urgency than high BMI levels (≥ 50 kg/m^2^). Indeed, our results show a significantly higher number of comorbidities, higher medication usage, CCI and ASA score, and a significantly lower estimated 10-year survival in patients with vs. without T2D and with vs. without CVD.

Our findings do not support using BMI ≥ 50 kg/m^2^ as a criterion to expedite access to bariatric and metabolic surgery, as we found no evidence of greater disease severity (as assessed by all variables used in this study) among patients with such level of BMI compared with patients with lower BMI.

These findings call for an urgent revision of existing criteria and practices in the NHS and other healthcare systems that recognize expedited access to surgery only in patients with BMI ≥ 50 kg/m^2^ [30]. Our data suggest that such practices may have detrimental consequences for many patients, as they effectively delay treatment among surgical candidates with lower BMI but at higher risk of disease-related complications and mortality.

The evolution from bariatric to metabolic surgery has important conceptual and practical ramifications for care and policy. While traditional bariatric surgery was conceived as a prophylactic treatment strategy aimed at the reduction of risk of obesity-related morbidity and mortality through weight loss, metabolic surgery is an intervention of therapeutic intent aimed at inducing remission or improvement of already established disease. This conceptually different approach, dictated by the type of baseline disease, warrants a radical overhaul of traditional care practices and policies of bariatric surgery.

The data from this study also provide an interesting perspective in the context of evolving treatment options for obesity. With the advent of more effective pharmacotherapy for obesity, particularly glucagon-like peptide (GLP-1) agonists and dual GLP-1/GIP (glucose-dependent insulinotropic polypeptide) agonists, questions have been raised about the role of surgery in the future treatment of obesity [31]. Although current FDA-approved pharmacotherapy for obesity can achieve clinically meaningful levels of initial weight loss, the long-term durability of weight reduction with pharmacotherapy remains unknown. In contrast, durable weight loss (ranging 20–30% from baseline) has been reported in studies with 10–20 years of follow-up after bariatric and metabolic surgery [27, 32]. Combined with the level-1 evidence from clinical trials documenting the ability of surgery to induce durable diabetes remission [27] and reduce diabetes-related complications and mortality [33], bariatric and metabolic surgery should still be considered the “gold standard” in patients with severe obesity (BMI > 35 kg/m^2^) and T2D.

Although many patients can benefit from a combination of lifestyle interventions and drug therapy, the management of multiple, severe obesity-related diseases will often require polypharmacy. As the number of medications required to effectively control risk factors and complications increases with diabetes and CVD status, so does the likelihood of adverse effects, drug-drug interactions, compliance problems and medication errors -thus increasing the overall risk and cost of therapy [34, 35]. Consequently, in patients with multiple/complex comorbidities, metabolic surgery may be a particularly suitable and more cost-effective treatment strategy compared to non-surgical management [36].

Despite its proven benefits, the uptake of bariatric/metabolic surgery remains low in most countries. Only 0.2–1% of patients who meet international guidelines criteria undergo bariatric and metabolic surgery [37]. Persistent weight bias and misconceptions surrounding the reversibility of obesity through lifestyle interventions still exist [38], even among physicians. Negative attitudes toward “weight loss surgery” among the general population create a difficult social environment for patients who opt for such types of treatment surgery [39]. Several studies have shown that metabolic surgery is more cost-effective [40] and is associated with greater reduction of CVD and overall mortality in patients with baseline diabetes compared with patients without diabetes [41, 42]. Despite such evidence, however, very few of these patients have access to bariatric/metabolic surgery; when they do, they are not generally considered for expedited access in most healthcare systems. Expedited access to surgical referral is in fact still based on BMI level [43]. In many healthcare systems, including the UK’s NHS, access to bariatric and metabolic surgery is often granted on a first-come, first-served basis rather than based on disease severity; when expedited access is recommended or granted, this is usually for surgical candidates with BMI ≥ 50 kg/m^2^. One practical barrier to transitioning away from legacy BMI-based criteria is the widespread reliance of health insurance systems, clinical pathways, and referral frameworks on BMI as a simple, easily measurable parameter, despite its limited correlation with actual disease burden [44, 45]. Additionally, existing institutional protocols and clinical commissioning policies typically lack robust mechanisms to dynamically evaluate comorbidity burden, further hindering the adoption of more sophisticated, disease-based triage models. Our findings clearly indicate that current practices are counterproductive for both individual patients and healthcare systems, as they can significantly delay necessary and urgent treatment. Future research should prioritize the development and validation of decision-making tools that integrate disease diagnosis and comprehensive metabolic risk profiles into prioritization frameworks. Additionally, evaluating the impact of these refined models on clinical outcomes, patient safety, and healthcare efficiency will be critical. Prospective multicenter studies combined with robust health-economic modeling could offer valuable evidence to facilitate large-scale policy shifts toward more clinically meaningful prioritization practices.

Our results underscore the urgency of revising current policies. Despite the increased operative risk observed in patients with T2D and CVD, our data demonstrate that bariatric and metabolic surgery remains safe in these higher-risk populations, with complication and readmission rates comparable to traditional bariatric procedures performed on lower-risk patients [46]. As an audit of routine clinical practice, this study has inherent limitations, including its retrospective design and dependence on a single-surgeon practice. Reflecting practice in a tertiary care level academic hospital, our series also has a greater prevalence of T2D and CVD compared to other types of bariatric and metabolic surgery practices. This means, however, that our study provides an opportunity to understand the implications of expanding indications for bariatric and metabolic surgery in patients with multiple/complex diseases at baseline, perhaps giving a valuable insight into the future landscape of the evolving field of bariatric and metabolic surgery.

In conclusion, our study demonstrates that current, widespread policies for prioritization of access to bariatric and metabolic surgery, exclusively based on BMI thresholds, do not reflect disease severity and, consequently, treatment urgency. Such practices are potentially harmful for patients and healthcare systems and should be urgently revised.

We strongly recommend the adoption of prioritization models that incorporate clinically relevant criteria, such as the presence of clinical obesity (as recently defined by the Lancet DE Commission [11]), T2D, or baseline CVD, as key indicators of urgency, rather than relying solely on BMI cut-offs.

Healthcare systems should consider establishing disease-based criteria for prioritization and/or triage frameworks based on more accurate measures of risk, accounting for multimorbidity and estimated survival projections, thereby enabling more equitable and timely surgical access for high-risk patients. These frameworks may also optimize resource allocation by reducing long-term costs associated with delayed treatment and complications of uncontrolled obesity-related diseases. This is especially relevant in light of recent evidence which emphasizes the superiority of surgical interventions in achieving sustained metabolic improvements and cardiovascular risk reduction, even when compared to modern pharmacotherapy [31, 47]. While advances in GLP-1 and GIP agonists are promising, long-term outcomes remain uncertain, and current data continue to position bariatric and metabolic surgery as the gold standard for treating severe obesity with complex multi-morbidity. Our findings add to a growing body of literature supporting a paradigm shift: from BMI-based criteria toward a more comprehensive, disease-based approach to access and reimbursement policies. This would ensure that the therapeutic intent of metabolic surgery is fully realized, targeting those who stand to benefit most, not just those who meet traditional BMI thresholds.

## Data Availability

No datasets were generated or analysed during the current study.

## Abbreviations

ASA: American society of anesthesiologists
BMI: Body mass index
BS: Bariatric surgery
CCI: Charlson comorbidity index
CKD: Chronic kidney disease
CVD: Cardiovascular disease
FR: Francesco Rubino
GLP-1: Glucagon-like peptide 1
HbA1c: Glycated haemoglobin A1c
LoS: Length of stay
MS: Metabolic surgery
NAFLD: Non-alcoholic fatty liver disease
NASH: Non-alcoholic steatohepatitis
MAFLD: Metabolic dysfunction-associated fatty liver disease
MASH: Metabolic dysfunction-associated steatohepatitis
NHS: National health service
SD: Standard deviation
T2D: Type 2 diabetes mellitus
WPW: Wolff–parkinson–white

## References

[1] Cummings DE, Rubino F. Diabetologia. 2018. 10.1007/s00125-017-4513-y. 10.1007/s00125-017-4513-y PMC6448954 29224190

[2] Rubino F, Marescaux J. Ann Surg. 2004. 10.1097/01.sla.0000102989.54824.fc. 10.1097/01.sla.0000102989.54824.fc PMC1356185 14685093

[3] Mingrone G, Panunzi S, et al. The Lancet. 2021. 10.1016/S0140-6736(20)32649-0.

[4] Syn NL, Cummings DE, et al. Lancet. 2021. 10.1016/s0140-6736(21)00591-2.

[5] Schiavon CA, Cavalcanti AB, et al. J Am Colleg Cardiol 2024. 10.1016/j.jacc.2023.11.032.

[6] Cohen RV, Pereira TV, et al. JAMA Surg 2020. 10.1001/jamasurg.2020.0420.

[7] Verrastro O, Panunzi S, et al. Lancet. 2023. 10.1016/s0140-6736(23)00634-7.

[8] Kovács G, Mohos E, et al. J Diabetes Res. 2023. 10.1155/2023/9686729. 10.1155/2023/9686729 PMC10748723 38144444

[9] Syn NL, Cummings DE, et al. The Lancet. 2021. PMCID. 10.1016/S0140-6736(21)00591-2.

[10] Rubino F, Shukla A, et al. Ann Surg. 2014. 10.1097/SLA.0b013e3182759656.

[11] Rubino F, Cummings DE, et al. Lancet Diabetes & Endocrinol. 10.1016/S2213-8587(24)00316-4.

[12] Romero-Corral A, Somers VK, et al. Int J Obes (Lond). 2008. 10.1038/ijo.2008.11.

[13] Sperrin M, Marshall AD, et al. J Pub Health 2016. 10.1093/pubmed/fdv067.

[14] Newson RS, Divino V, et al. Diabetes Therapy 2024. 10.1007/s13300-024-01583-w. 10.1007/s13300-024-01583-w PMC11096291 38683494

[15] Yu T, Wong T-J, et al. Obesity Research & Clinical Practice. 2024. PMCID. 10.1016/j.orcp.2024.02.002. 10.1016/j.orcp.2024.02.002 38331596

[16] Obesity: identification, assessment and management. London: National Institute for Health and Care Excellence (NICE); 2023. 36719951

[17] Rubino F, Cohen RV, et al. Lancet Diabetes Endocrinol. 2020. 10.1016/s2213-8587(20)30157-1.

[18] ElSayed NA, Aleppo G, et al. Diabetes Care. 2023. 10.2337/dc23-ad08.

[19] Mach F, Baigent C, et al. Eur Heart J 2020. 10.1093/eurheartj/ehz455.

[20] Saklad M. Grading of patients for surgical procedures. Anesthesiology. 1941;2(3):281–4. 10.1097/00000542-194105000-00004

[21] Dripps RD. New clas-sification of physical status. Anesthesiology. 1963;24:111.

[22] Charlson ME, Pompei P, et al. J Chronic Dis. 1987. 10.1016/0021-9681(87)90171-8.

[23] Huang YQ, Gou R, et al. J Zhejiang Univ Sci B 2014. 10.1631/jzus.B1300109.

[24] Dindo D, Demartines N, Clavien PA. Ann Surg. 2004. 10.1097/01.sla.0000133083.54934.ae. 10.1097/01.sla.0000133083.54934.ae PMC1360123 15273542

[25] Courcoulas A, Coley RY, et al. JAMA Surg. 2020. 10.1001/jamasurg.2019.5470.

[26] Buchwald H, Avidor Y, et al. Jama. 2004. 10.1001/jama.292.14.1724. 10.1001/jama.292.14.1724 15479938

[27] Courcoulas AP, Patti ME, et al. JAMA 2024. 10.1001/jama.2024.0318.

[28] Kirwan JP, Courcoulas AP, et al. Diabetes Care. 2022. 10.2337/dc21-2441. 10.2337/dc21-2441 PMC9490448 35320365

[29] Fisher DP, Johnson E, et al. JAMA. 2018. 10.1001/jama.2018.14619.

[30] German Society for General and Visceral Surgery (DGAV), German Obesity Society (DAG), German Diabetes Association (DDG), et al. S3 Guideline: Surgery of Obesity and Metabolic Diseases. Leitlinienprogramm der DGAV. Version 1.0, 2018. Available from: https://www.awmf.org/leitlinien/detail/ll/088-001.html

[31] Pipek LZ, Moraes WAF, et al. Sci Rep. 2024. 10.1038/s41598-024-57724-5.

[32] O’Brien PE, Hindle A, et al. Obes Surg*.* 2019. 10.1007/s11695-018-3525-0.

[33] Welbourn R, Hollyman M, et al. Obes Surg. 2019. 10.1007/s11695-018-3593-1.

[34] Rollason V, Vogt N. Drugs Aging*.* 2003. 10.2165/00002512-200320110-00003. 10.2165/00002512-200320110-00003 12964888

[35] Grundy SM. Nat Rev Drug Discov 2006. 10.1038/nrd2005. 10.1038/nrd2005 16582875

[36] Borisenko O, Lukyanov V, Ahmed AR. Br J Surg*.* 2018. 10.1002/bjs.10857. 10.1002/bjs.10857 29667178

[37] Martin M, Beekley A, et al. Surg Obes Relat Dis 2010. 10.1016/j.soard.2009.07.003. 10.1016/j.soard.2009.07.003 19782647

[38] O’Keeffe M, Flint SW, et al. Lancet Diabetes Endocrinol. 2020. 10.1016/s2213-8587(20)30073-5.

[39] Dolan P, Afaneh C, et al. JAMA Surg. 2019. 10.1001/jamasurg.2018.4650.

[40] Lauren BN, Lim F, et al. JAMA Network Open. 2022. 10.1001/jamanetworkopen.2021.48317.

[41] Eliasson B, Liakopoulos V, et al. Lancet Diabetes & Endocrinol 2015. 10.1016/S2213-8587(15)00334-4.

[42] Aminian A, Zajichek A, et al. Jama 2019. 10.1001/jama.2019.14231.

[43] Livingston EH. Jama 2012. 10.1001/jama.2011.1950.

[44] Sweatt K, Garvey WT, Martins C. Curr Obes Rep. 2024. 10.1007/s13679-024-00580-1. 10.1007/s13679-024-00580-1 PMC11306271 38958869

[45] Nuttall FQ. Nutr Today. 2015. 10.1097/nt.0000000000000092. 10.1097/NT.0000000000000092 PMC4890841 27340299

[46] Chang S-H, Stoll CRT, et al. JAMA Surg. 2014. 10.1001/jamasurg.2013.3654.

[47] Ikramuddin S, Korner J, et al. JAMA. 2018. 10.1001/jama.2017.20813.

